# Synthesis and Evaluation of Phenylxanthine Derivatives as Potential Dual A2AR Antagonists/MAO-B Inhibitors for Parkinson’s Disease

**DOI:** 10.3390/molecules22061010

**Published:** 2017-06-17

**Authors:** Xuebao Wang, Chao Han, Yong Xu, Kaiqi Wu, Shuangya Chen, Mangsha Hu, Luyao Wang, Yun Ye, Faqing Ye

**Affiliations:** School of Pharmaceutical Sciences, Wenzhou Medical University, Wenzhou 325035, China; wangxuebao@wmu.edu.cn (X.W.); lab1509@163.com (C.H.); x13766995321@163.com (Y.X.); k13578902233@163.com (K.W.); dsd1801@163.com (S.C.); m199720@sina.com (M.H.); w1386565198@163.com (L.W.); 13957720727@163.com (Y.Y.)

**Keywords:** adenosine A2AR antagonist, monoamine oxidase B inhibitor, phenylxanthine derivatives, parkinson′s disease

## Abstract

The aim of this research was to prove the speculation that phenylxanthine (PX) derivatives possess adenosine A2A receptor (A2AR)-blocking properties and to screening and evaluate these PX derivatives as dual A2AR antagonists/MAO-B inhibitors for Parkinson′s disease. To explore this hypothesis, two series of PX derivatives were prepared and their antagonism against A2AR and inhibition against MAO-B were determined in vitro. In order to evaluate further the antiparkinsonian properties, pharmacokinetic and haloperidol-induced catalepsy experiments were carried out in vivo. The PX-D and PX-E analogues acted as potent A2AR antagonists with Ki values ranging from 0.27 to 10 μM, and these analogues displayed relatively mild MAO-B inhibition potencies, with inhibitor dissociation constants (Ki values) ranging from 0.25 to 10 μM. Further, the compounds **PX-D-P6** and **PX-E-P8** displayed efficacious antiparkinsonian properties in haloperidol-induced catalepsy experiments, verifying that these two compounds were potent A2AR antagonists and MAO-B inhibitors. We conclude that PX-D and PX-E analogues are a promising candidate class of dual-acting compounds for treating Parkinson′s disease.

## 1. Introduction

Parkinson′s disease (PD) is the second most common progressive neurodegenerative disorder after Alzheimer's disease and it affects approximately 1% of the population over the age of 65 [1,2]. PD is primarily caused by dysfunction of dopaminergic neurons, and the therapy of PD is largely centered on dopamine replacement strategies with the dopamine agonist and dopamine precursor levodopa drugs [3]. Ongoing studies have shown new insights regarding the pathophysiology of PD, which suggest that the non-dopaminergic system is also affected and may correlate with multiple PD symptoms [4,5]. Non-dopaminergic targets are potentially used to reduce levodopa dosage or to treat non-levodopa-responsive symptoms.

A2AR is not widely distributed in the central nervous system, but rather it is located in selective areas of the brain. It is expressed richly in the nigrostriatum, where it co-localizes with dopamine D2 receptors on output neurons [6]. Besides, A2AR is also located in glutamatergic terminals and controls the release of glutamate from afferent glutamatergic terminals which trigger the activity in the striatum, and A2AR is highly important in the control of voluntary movement [7,8,9], so a lot of research groups have dedicated significant efforts towards the discovery of selective A2AR antagonists for the therapy of PD [10]. Istradefylline was the first A2AR antagonist which was evaluated as an adjunctive therapy with levodopa with motor fluctuations in PD patients. In 2013, Istradefylline was licensed in Japan, because of its positive results and a further multicenter Phase III trial is ongoing in the U.S. [11]. Moreover, several Phase III studies (with drugs such as preladenant, tozadenant and caffeine) have already been conducted with positive results [10]. In a word, A2AR antagonists represent hopeful adjunctives to dopamine replacement treatment for PD.

**Figure 1:**
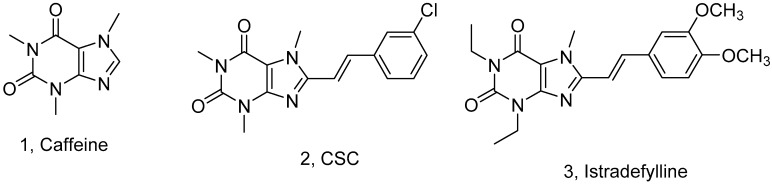
The structures of adenosine A2AR antagonists caffeine (**1**), CSC (**2**), and istradefylline (**3**).

**Figure 2:**
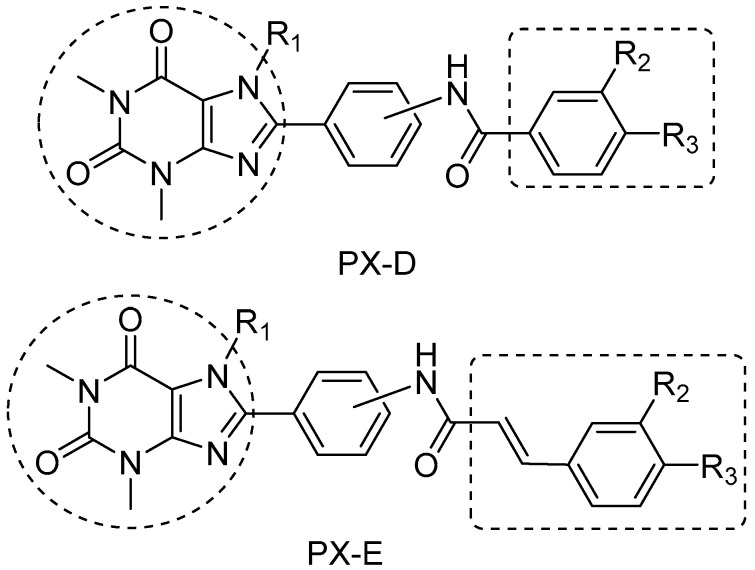
Parent nucleus for the target compounds of the PX-D and PX-E series.

**Figure 3:**
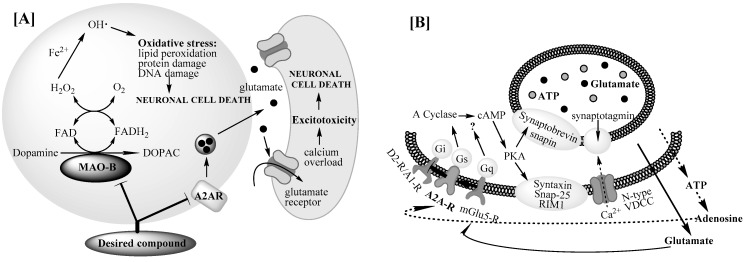
(**A**) Dual molecular mechanism of the A2AR antagonists/MAO-B inhibitors. Desirable compounds will prevent neuron death by antioxidant effects via MAO-B inhibition and prevention of excitotoxic release of glutamate via A2AR antagonism. (**B**) Schematic instruction of glutamate release by the ability of A2A receptors: strong cortico-limbic-thalamic input facilitate A2A and mGlu5 synergistic activation results in increases in the probability of glutamate release.

In our previous studies [12,13], we have reported that a group of PX derivatives are potent inhibitors of monoamine oxidase B (MAO-B) which are known to possess antiparkinsonian properties [14,15]. Since xanthine derivatives such as caffeine, CSC and istradefylline (Figure 1) have the ability to block A2AR, we speculated that PX derivatives (Figure 2) might also have similar ability. A similar idea had been verified with the compound 8-(3-chlorostyryl) caffeine [16]. The aim of this research was to explore the speculation that PX derivatives possess antiparkinsonian properties and to screen and evaluate these PX derivatives as dual A2AR antagonists/MAO-B inhibitors for treatment of Parkinson′s disease. The dual molecular mechanism of the possible A2AR antagonists/MAO-B inhibitors is shown in Figure 3 [8,17].

## 2. Results and Discussion

### 2.1. Chemistry

**Scheme 1:**
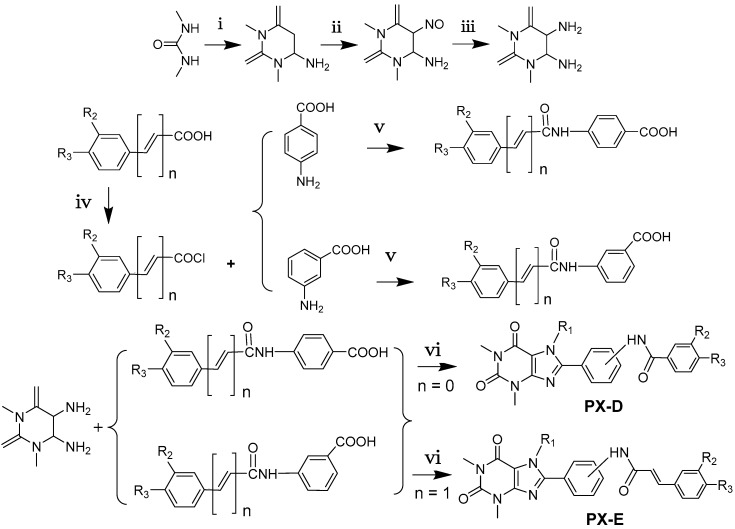
General method for the synthesis of phenylxanthine derivatives of series PX-D and PX-E, *n* = 0 or 1.

Scheme 1 shows the general route for the synthesis of phenylxanthine derivatives of series PX-D and PX-E. The compounds were synthesized with yields between 40.7–56.3% following a six-step procedure.

### 2.2. Structure−Activity Relationship between Test Compounds and A2AR

Initially, a series of PX derivatives were analysed with the objective of screening novel, potent and selective A2AR antagonists. The data obtained are listed in Table 1. 

**Figure 4:**
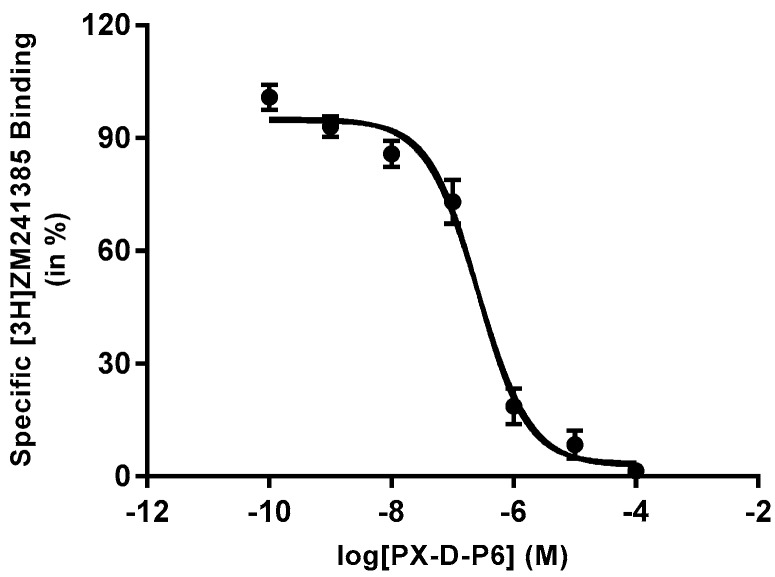
The sigmoidal concentrations–response curve of specific [3H]ZM241385 binding at human A2AR expressed in HEK293. The A2AR proteins were incubated with the radiolabelled compound for 30 min and exposed to different concentrations (10^−4^, 10^−5^, 10^−6^, 10^−7^, 10^−8^, 10^−9^, 10^−10^ M) of the compound **PX-D-P6**. Results are expressed as the percentage of the control binding. The meaning of 100% is that A2A receptors of incubating membranes are all bonded with [3H]ZM241385, and binding rate of test compounds close to zero. The experiments were performed in duplicate and the values are displayed as mean ± SD.

Most of the PX derivatives showed good binding affinity, with Ki values in the range of 0.27–10 μM for human A2AR. In general, PX-D derivatives showed better binding affinity than PX-E derivatives; *para*-substituents on the phenylxanthine with -HNCO moieties lead to better activity than *meta*-substituents. Methyl substituents on the R_1_ position are more favorable than hydrogen atoms; on the R_2_ and R_3_ positions, electron-donating CH_3_ or -OCH_3_ groups showed poorer Ki values than -H or electron-withdrawing groups like -Cl or -F, except for the stronger electron-withdrawing group -CF_3_. In addition, when both of the R_2_ and R_3_ positions contained -OCH_3_, the Ki values are generally poor. The larger electron withdrawing effect and steric hindrance between test compounds and A2AR may be the reason for the different affinity. The potencies of the test compounds antagonizing A2AR were assayed by radioligand binding experiments [18]. The concentration–response curve for the test compounds versus [3H]ZM241385 are exemplified with **PX-D-P6** (Figure 4). This compound was picked out as one of the potent antagonists, with a Ki value of 0.33 ± 0.07 μM.

### 2.3. Structure−Activity Relationship between Compounds and MAO-B

As shown in Table 1, most of the PX derivatives were found to exhibit better MAO-B inhibition activity than that of istradefylline, especially **PX-D-P4**, which showed a better selectivity with an inhibition rate of 0.25 μM (Ki value). Concerning the structures, PX-D analogues showed better inhibitory potencies than PX-E analogues. 

*para*-Substituents on the phenylxanthine with -HNCO show better inhibition activity than *meta*-substituents. When both of the R_2_ and R_3_ position were occupied by -OCH_3_, the Ki values are generally poor. There was no significant rule about different substituents in the R_1_, R_2_ and R_3_ positions, regarding the MAO-B inhibition activity. Perhaps the steric hindrance played a key role in the selectivity of MAO-B inhibition.

**Figure 5:**
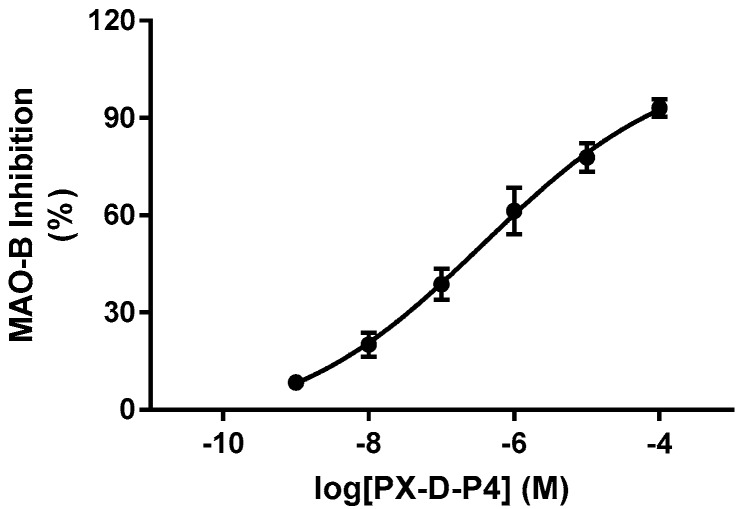
Inhibition curve of compound PX-D-P4 on MAO-B activity. The IC_50_ value was calculated by fitting the data, using nonlinear least-squares regression analysis. Results are expressed as the percentage of the MAO-B activity inhibition. The meaning of 100% is that MAO-B activity has been inhibited to the lowest activity for converting luminogenic MAO-B substrate to luciferin, which can produce light, and the produced light intensity is near to the level of the substrate absence. The experiments were performed in duplicate and the values are displayed as mean ± SD.

The MAO-B inhibitory activity of the test compounds was determined by a fluorometric method with a commercial kit procedure [18]. An example of the data obtained for the IC_50_ determinations is illustrated for **PX-D-P4** (Figure 5). The Ki values are obtained from the measured IC_50_ values, which were calculated by the Cheng-Prusoff equation [19].

### 2.4. Cell Cytotoxicity

**Figure 6:**
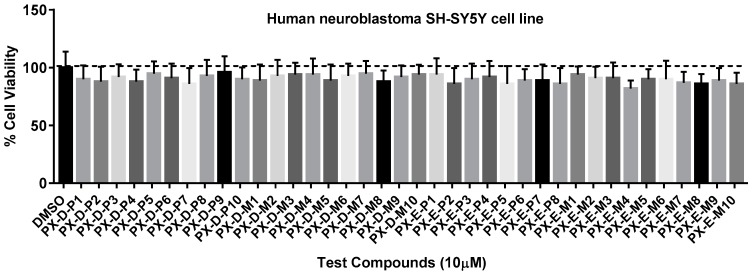
Cytotoxicity of test compounds against human neuroblastoma SH-SY5Y cell line was measured by the MTT method after 24 h. The values are displayed as mean ± SD.

Human neuroblastoma SH-SY5Y cell line was chosen for testing the cell cytotoxicity, because it had been used as an in vitro model of neuronal function and differentiation. SH-SY5Y cells were exposed to relatively high concentrations of test compounds, in order to observe the cell cytotoxicity. The antiproliferative activity results all test compounds are shown in Figure 6 in the form of mean ± SD. The % cell viability values were more than 88%. The results indicate that cytotoxicity of test compounds to SH-SY5Y cells is very low. Moreover, test compounds with different structure have little discrepancy in antiproliferative activities. 

### 2.5. Pharmacokinetics and Brain Distribution of PX-D-P6 and PX-E-P8

According to the biological data of A2AR affinities and MAO-B inhibitory potencies, **PX-D-P6** and **PX-E-P8** were chosen for further efficacy studies in vivo. Pharmacokinetics and brain distribution were studied as a matter of priority in order to observe their absorption, elimination and brain penetration. The primary pharmacokinetic parameter results of **PX-D-P6** and **PX-E-P8** are summarized in Table 2. 

The T_max_ (time of peak plasma concentration) after i.p. administration was 0.51 h and 0.48 h for compounds **PX-D-P6** and **PX-E-P8**, respectively. The t_1/2_ (half-life) was 4.51 h and 3.27 h for **PX-D-P6** and **PX-E-P8**, respectively. The results that plasma and brain assays for **PX-D-P6** and **PX-E-P8** at 1 h after i.p. 50 mg/kg dosing in rats are shown in Table 3. Compounds **PX-D-P6** and **PX-E-P8** both displayed the capacity to cross the blood-brain barrier (brain/plasma ratio = 1.14 and 0.97 for **PX-D-P6** and **PX-E-P8**, respectively), which is an important property for the treatment of Parkinson′s disease. Compared to **PX-E-P8**, compound **PX-D-P6** had better pharmacokinetic parameters and brain penetration.

### 2.6. Haloperidol Induced Catalepsy Study

**Figure 7:**
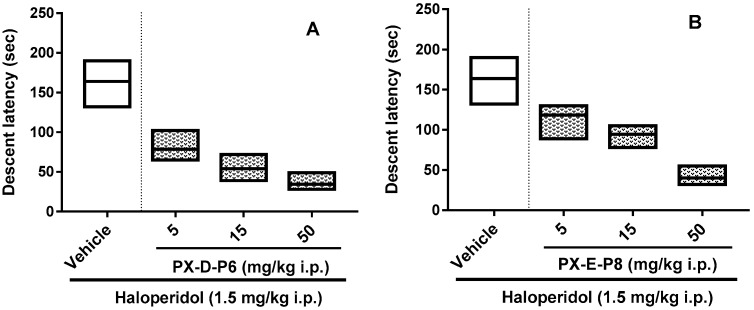
Effects of PX-D-P6 (**A**) and PX-E-P8 (**B**) on haloperidol-induced catalepsy in rats with different concentrations (5 mg/kg, 15 mg/kg, 50 mg/kg). Each column indicates the median and interquartile ranges, and the number of rats used was six, *p* < 0.05 (Bonferroni's test), compared with the vehicle-treated group.

Haloperidol blocks the nigrostriatal dopamine transmission leading to symptoms such as catalepsy and muscular rigidity, making it a robust animal model for screening of antiparkinsonian drugs [20]. Selective antagonists of A2AR were reported to resist the catalepsy induced by haloperidol [21,22]. In the chronic haloperidol model, treatment with different doses of **PX-D-P6** and **PX-E-P8** (5, 15 and 50 mg/kg) was administered. The effects of **PX-D-P6** (A) and **PX-E-P8** (B) on haloperidol-induced catalepsy in rats are displayed in Figure 7. On the whole, the effect of both **PX-D-P6** and **PX-E-P8** was a significant reduction in catalepsy with different doses. Figure 7A shows that there was a dose-dependent relationship between compound **PX-D-P6** and reduction in catalepsy, and **PX-D-P6** displayed significant anti-catalepsy ability at a dose of 50 mg/kg. Only with high dose, could compound **PX-E-P8** display significant anti-catalepsy ability, and it had slight effects at low dose (Figure 7B). 

The anti-catalepsy efficacy between **PX-D-P6** and **PX-E-P8** was different at low dose. The reason could derive from the differences in biological data (**PX-D-P6**: Ki hA2AR, 330 nM; Ki hMAO-B, 290 nM; **PX-E-P8**: Ki hA2AR, 850 nM; Ki hMAO-B, 630 nM), and from the different B/P ratios (**PX-D-P6**: 1.14; **PX-E-P8**: 0.97). These differences resulted in low brain concentration of **PX-E-P8**, so that the anti-catalepsy efficacy was unsatisfactory with a low dose. 

Comparing their structures, **PX-E-P8** has a larger electron withdrawing effect and steric hindrance, which could reduce the affinity with A2A receptor and increase the difficulty in penetrating the blood brain barrier.

Comparing with other reported dual-acting compounds for Parkinson′s disease, The anti-catalepsy efficacy of compounds **PX-D-P6** and **PX-E-P8** is slightly better than that of compound **17f** reported by Rivara in 2013 [18]. In this study, the result was that “compound **17f** significantly antagonized haloperidol-induced catalepsy at doses of 30 and 100 mg/kg, while it was inactive at a dose of 10 mg/kg”.

## 3. Materials and Methods 

### 3.1. Materials

All organic chemicals were available commercially and used without further purification. The common reagents were purchased from Sinopharm Chemical Reagent Corporation (Shanghai, China), except for monoamine oxidase B (Sigma-Aldrich Corporation, St. Louis, Missouri, USA), and [3H]ZM241385 (Biotrend, Cologne, Germany). Thin layer chromatography was performed on silica gel plates (F254, 0.2 mm thick). The IR spectra were assayed on a Nicolet NEXUS 670 FT-IR Spectrometer in KBr pellets (Thermo Scientific Corporation, Waltham, Massachusetts, USA). Mass spectra were recorded with a Bruker Esquire HCT plus mass spectrometer in the positive ion mode (Bruker Corporation, Ettlingen, Germany). ^1^H nuclear magnetic resonance spectra were recorded on a Bruker AVANCE III 600 MHz NMR spectrometer (Bruker Corporation, Ettlingen, Germany). The splitting patterns of NMR spectra were designated as follows: s: singlet; d: doublet; t: triplet; m: multiplet; *J*: coupling constants. Elemental analysis for C, H and N was performed using a Flash EA 1112 Elemental Analyzer (Thermo Scientific Corporation) and were found within ± 0.5% of the theoretical values. The bound radioactivity for A2AR binding experiments was assayed by a MicroBeta counter (PerkinElmer Corporation, Fremont, California, USA). The luminescent signal for MAO-B activity assay was read at a GloMax 96 Microplate Luminometer (Promega Corporation, Madison, Wisconsin, USA). 

### 3.2. Synthesis of PX-D and PX-E Analogues

The general route design for the synthesis of phenylxanthine derivatives of the PX-D and PX-E series was shown in Scheme 1. For details of the synthesis of **PX-D-P4**, **PX-D-P5**, **PX-D-P9**, **PX-D-P10**, **PX-D-M4**, **PX-D-M55**, **PX-D-M9**, **PX-D-M10**, **PX-E-P4, PX-E-P8**, **PX-E-M5**, **PX-E-M10**, see our previously reported analogues [10,11]. The other compounds had been reported in two earlier articles [10,11]. Structural character­ization data (FT-IR, ^1^H-NMR, mass spectra and elemental analysis) of all the synthesized PX-D and PX-E analogues was as follows:

*3-Chloro-N-(4-(1,3-dimethyl-2,6-dioxo-2,3,6,7-tetrahydro-1H-purin-8-yl)phenyl)benzamide* (**PX-D-P4**). Dark brown solid, Yield: 58.2%; mp > 300 °C; IR (cm^−1^): ν_N-H_: 3289.12, 3175.20; ν_C=O_: 1697.67, 1678.72, 1662.48; ^1^H-NMR (DMSO-d_6_): 3.27 (s, 3H, N-CH_3_), 3.51 (s, 3H, N-CH_3_), 7.60 (t, 1H, *J* = 7.8 Hz, 5′′-NHCO-PhH), 7.695 (d, 1H, *J* = 8.4 Hz, CONH-PhH), 7.93 (m, 3H, CONH-PhH + 4′′,6′′-NHCO-PhH), 8.02 (s,1H, 2′′-NHCO-PhH), 8.14 (d, 2H, *J* = 8.4 Hz, CONH-PhH × 2), 10.56 (s, 1H, CONH), 13.73 (s, 1H, xanthineH). EI-MS: 410.1 [M + H]^+^. Chemical Formula: C_20_H_16_ClN_5_O_3_, Calcd. For: C, 58.61; H, 3.94; N, 17.09. Found: C, 58.52; H, 3.95; N, 17.06. 

*N-(4-(1,3-Dimethyl-2,6-dioxo-2,3,6,7-tetrahydro-1H-purin-8-yl)phenyl)-3,4-dimethoxybenzamide* (P**X-D-P5**). Dark brown solid, Yield: 49.8%; mp > 300 °C; IR (cm^−1^): ν_N–-H_: 3464.56, 3149.94; ν_C=O:_ 1678.84, 1670.30, 1662.19. ^1^H-NMR (DMSO-d_6_): 3.27(s, 3H, N-CH_3_), 3.51(s, 3H, N-CH_3_), 3.85 (s, 6H, OCH_3_ × 2), 7.11 (d, 1H, *J* = 8.4 Hz, CONH-PhH), 7.54 (d, 1H, *J* = 7.8 Hz, NHCO-PhH), 7.65 (s, 1H, 2′-NHCO-PhH), 7.86 (d, 1H, *J* = 7.8 Hz, NHCO-PhH), 7.92 (m, 1H, CONH-PhH), 8.13 (m, 2H, CONH-PhH × 2), 10.29 (s, 1H, CONH), 13.73 (s, 1H, xanthineH). EI-MS: 436.0 [M + H]^+^. Chemical Formula: C_22_H_21_N_5_O_5_; Calcd. For: C, 60.68; H, 4.86; N, 16.08; Found: C, 60.67; H, 4.81; N, 16.11.

*3-Chloro-N-(4-(1,3,7-trimethyl-2,6-dioxo-2,3,6,7-tetrahydro-1H-purin-8-yl)phenyl)benzamide* (**PX-D-P9**). Dark brown solid, Yield: 53.4%; mp > 300 °C; IR (cm^−1^): ν_N–H_: 3381.99; ν_C=O_: 1666.59, 1649.87. ^1^H-NMR (DMSO-d_6_): 3.27 (s, 3H, N-CH_3_), 3.38 (s, 3H, N-CH_3_), 4.03 (s, 3H, N-CH_3_), 7.62 (t, 1H, *J* = 7.8 Hz, 5′-NHCO-PhH), 7.71 (d, 1H, *J* = 6.6 Hz, NHCO-PhH), 7.85 (d, 2H, *J* = 9 Hz, CONH-PhH × 2), 7.95 (d, 1H, *J* = 7.8 Hz, NHCO-PhH), 8.00 (d, 2H, *J* = 9 Hz, CONH-PhH × 2), 8.04 (s, 1H, 2′-NHCO-PhH), 10.61 (s, 1H, CONH). EI-MS: 424.0 [M + H]^+^. Chemical Formula: C_21_H_18_ClN_5_O_3_; Calcd. For: C, 59.51; H, 4.28; N, 16.52; Found: C, 59.54; H, 4.30; N, 16.56.

*3,4-Dimethoxy-N-(4-(1,3,7-trimethyl-2,6-dioxo-2,3,6,7-tetrahydro-1H-purin-8-yl)phenyl)benzamide* (**PX-D-P10**). Dark brown solid, Yield: 47.5%; mp > 300 °C; IR (cm^-1^): ν_N-H_: 3527.37, 3204.87; ν_C=O_: 1701.45, 1688.34, 1658.48. ^1^H-NMR (DMSO-d_6_): 3.28 (s, 3H, N-CH_3_), 3.35 (s, 3H, N-CH_3_), 3.38 (s, 3H, N-CH_3_), 3.52 (s, 3H,-OCH_3_), 3.83 (s, 3H, -OCH_3_), 7.04 (d, 2H, *J* = 8.4 Hz, CONH-PhH × 2), 7.22 (d, 1H, *J* = 7.8 Hz, NHCO-PhH), 7.46 (s, 1H, 2′′-NHCO-PhH), 7.54 (d, 1H, *J* = 7.8 Hz, NHCO-PhH), 7.84 (d, 2H, *J* = 8.4 Hz,CONH-PhH × 2), 10.33 (s, 1H, CONH). EI-MS: 450.0 [M + H]^+^. Chemical Formula: C_23_H_23_N_5_O_5_; Calcd. For: C, 61.46; H, 5.16; N, 15.58; Found: C, 61.49; H, 5.13; N, 15.52.

*3-Chloro-N-(3-(1,3-dimethyl-2,6-dioxo-2,3,6,7-tetrahydro-1H-purin-8-yl)phenyl)benzamide* (**PX-D-M4**). Dark brown solid, Yield: 55.6%; mp > 300 °C; IR (cm^-1^): ν_N-H_: 3268.69; ν_C=O_: 1701.17, 1638.11, 1659.97. ^1^H-NMR (DMSO-d_6_): 3.25 (s, 3H, N-CH_3_), 3.45 (s, 3H, N-CH_3_), 7.355 (d, 2H, *J* = 7.8 Hz, CONH-PhH × 2), 7.486 (t, 1H, *J* = 15.6 Hz, 5′′-NHCO-PhH), 7.886 (m, 2H, CONH-PhH × 2), 7.921 (d, 2H, *J* = 15.6 Hz, 4′,6′-NHCO-PhH), 8.568 (s, 1H, 2′-NHCO-PhH), 10.29 (s, 1H, CONH), 13.90 (s, 1H, xanthineH). EI-MS: 409.8 [M + H]^+^. Chemical Formula: C_20_H_16_ClN_5_O_3_, Calcd. For: C, 58.61; H, 3.94; N, 17.09; Found: C, 58.57; H, 3.90; N, 17.12.

*N-(3-(1,3-Dimethyl-2,6-dioxo-2,3,6,7-tetrahydro-1H-purin-8-yl)phenyl)-3,4-dimethoxybenzamide* (**PX-D-M5**). Dark brown solid, Yield: 50.9%; mp > 300 °C; IR (cm^−1^): ν_N-H_: 3502.43 ; ν_C=O_: 1701.42, 1674.42, 1664.52. ^1^H-NMR (DMSO-d_6_): 3.28 (s, 3H, N-CH_3_), 3.39 (s, 3H,N-CH_3_), 3.53 (s, 3H, -OCH_3_), 3.86 (s, 3H, -OCH_3_), 7.11 (d, 2H, J = 8.4 Hz, CONH-PhH), 7.50 (t, 1H, *J* = 7.8 Hz, CONH-PhH), 7.670 (s, 1H, CONH-PhH), 7.89 (m, 2H, NHCO-PhH × 2), 8.52 (s, 1H, 2′-NHCO-PhH), 10.28 (s, 1H, CONH), 13.91 (s, 1H, xanthineH). EI-MS: 436.0 [M + H]^+^. Chemical Formula: C_22_H_21_N_5_O_5_; Calcd. For: C, 60.68; H, 4.86; N, 16.08; Found: C, 60.65; H, 4.81; N, 16.09.

*3-Chloro-N-(3-(1,3,7-trimethyl-2,6-dioxo-2,3,6,7-tetrahydro-1H-purin-8-yl)phenyl)benzamide* (**PX-D-M9**). Dark brown solid, Yield: 56.0%; mp > 300 °C; IR (cm^−1^): ν_N-H_: 3248.75,3156.49; ν_C=O_: 1704.37, 1679.88, 1675.25. ^1^H-NMR (DMSO-d_6_): 3.24 (s, 3H, N-CH_3_), 3.46 (s, 3H, N-CH_3_), 4.02 (s, 3H, N-CH_3_), 7.54 (d, 2H, *J* = 6.6Hz, 4′,6′-NHCO-PhH × 2), 7.57 (t, 1H, *J* = 7.8 Hz, CONH-PhH), 7.67 (d, 1H, *J* = 7.8, CONH-PhH), 7.93 (d, 1H, *J* = 8.4 Hz, CONH-PhH), 7.96 (t, 1H, *J* = 6.6 Hz, 5′-NHCO-PhH), 8.03 (s, 1H, CONH-PhH), 8.20 (s, 1H, 2′-NHCO-PhH), 10.55 (s, 1H, CONH). EI-MS: 424.2 [M + H]^+^. Chemical Formula: C_21_H_18_ClN_5_O_3_; Calcd. For: C, 59.51; H, 4.28; Cl, 8.36; N, 16.52; Found: C, 59.45; H, 4.30; N, 16.55.

*3,4-Dimethoxy-N-(3-(1,3,7-trimethyl-2,6-dioxo-2,3,6,7-tetrahydro-1H-purin-8-yl)phenyl)benzamide* (**PX-D-M10**). Dark brown solid, Yield: 56.3%; mp > 300 °C; IR (cm^−1^): ν_N-H_: 3505.78; ν_C=O_: 1697.61, 1657.12. ^1^H-NMR (DMSO-d_6_): 3.27 (s, 3H, N-CH_3_), 3.49 (s, 3H,N-CH_3_), 3.85 (s, 3H, N-CH_3_), 3.86 (s, 3H, -OCH_3_), 4.04 (s, 3H, -OCH_3_), 7.11 (s, 1H, PhH), 7.54 (m, 2H, PhH), 7.56 (m, 1H, PhH), 7.66 (d, 1H, *J* = 7.8 Hz, NHCO-PhH), 7.92 (d, 1H, *J* = 7.8 Hz, NHCO-PhH), 8.23 (s, 1H, 2′-NHCO-PhH), 10.43 (s, 1H, CONH). EI-MS: 450.0 [M + H]^+^. Chemical Formula: C_23_H_23_N_5_O_5_, Calcd. For: C, 61.46; H, 5.16; N, 15.58; Found: C, 61.37; H, 5.13; N, 15.64

*(E)-3-(3-Chlorophenyl)-N-(4-(1,3-dimethyl-2,6-dioxo-2,3,6,7-tetrahydro-1H-purin-8-yl)phenyl)acrylamide* (**PX-E-P4**). Dark brown solid, Yield: 46.5%; mp > 300 °C; IR (cm^−1^): ν_N-H_: 3467.64, 3126.09. ν_C=O_: 1687.37, 1678.34, 1673.89. ^1^H-NMR (DMSO-d_6_): 3.27 (s, 3H,N-CH_3_), 3.51 (s, 3H,N-CH_3_), 6.91 (t, 1H, *J* = 15.6 Hz, 5′-PhH), 7.62 (d, 2H, *J* = 15.6 Hz, 4′,6′-PhH), 7.49 (m, 2H, = CH × 2), 7.71 (d, 2H, *J* = 9.0 Hz, CONH-PhH × 2), 7.83 (s, 1H, 2′-PhH), 8.11 (d, 2H, *J* = 8.4 Hz, CONH-PhH × 2), 10.47 (s, 1H, CONH), 13.73 (s, 1H, xanthineH). EI-MS: 436.1 [M + H]^+^. Chemical Formula: C_22_H_18_ClN_5_O_3;_ Calcd. For: C, 60.62; H, 4.16; N, 16.07; Found: C, 60.51; H, 4.19; N, 16.02

*(E)-3-(3-Chlorophenyl)-N-(4-(1,3,7-trimethyl-2,6-dioxo-2,3,6,7-tetrahydro-1H-purin-8-yl)phenyl)acrylamide* (**PX-E-P8**). Dark brown solid, Yield: 42.3%; mp > 300 °C; IR (cm^−1^): ν_N-H_: 3508.03; ν_C=O_: 1686.59, 1644.26. ^1^H-NMR (DMSO-d_6_): 3.26 (s, 3H, N-CH_3_), 3.48 (s, 3H, N-CH_3_), 4.028 (s, 3H, N-CH_3_), 6.93 (t, 1H, *J* = 15.6 Hz, 5′-PhH), 7.50 (m, 2H, = CH × 2), 7.62 (d, 2H, *J* = 8.4Hz, CONH-PhH × 2), 7.73 (s, 1H, 2′-PhH), 7.83 (d, 2H, *J* = 8.4 Hz, CONH-PhH × 2), 7.89 (d, 2H, *J* = 9.0 Hz, 4′,6′-PhH), 10.54 (s, 1H, CONH). EI-MS: 450.1 [M + H]^+^. Chemical Formula: C_23_H_20_ClN_5_O_3_; Calcd. For: C, 61.40; H, 4.48; N, 15.57; Found: C, 61.51; H, 4.47; N, 15.63.

*(E)-3-(3,4-Dimethoxyphenyl)-N-(4-(1,3-dimethyl-2,6-dioxo-2,3,6,7-tetrahydro-1H-purin-8-yl)phenyl)-acrylamide* (**PX-E-M5**). Dark brown solid, Yield: 40.7%; mp > 300 °C; IR (cm^-1^): ν_N-H_: 3340.88, 3305.87; ν_C=O_: 1697.77, 1650.21, 1630.65. ^1^H-NMR (DMSO-d_6_): 3.28 (s, 3H, N-CH_3_), 3.52 (s, 3H, N-CH_3_), 3.81 (s, 3H, -OCH_3_), 3.83 (s, 3H, -OCH_3_), 6.74 (d, 1H, *J* = 15.6 Hz, 6′-PhH), 7.57 (d, 1H, *J* = 15.6 Hz, 5′-PhH), 7.04 (d, 1H, *J* = 8.4 Hz, CONH-PhH), 7.22 (m, 2H, = CH × 2), 7.46 (t, 1H, *J* = 8.4 Hz, CONH-PhH), 7.84 (m, 2H, CONH-PhH), 8.44 (s, 1H, 2′-PhH), 10.34 (s, 1H, CONH), 13.96 (s, 1H, xanthineH). EI-MS: 462.0 [M + H]^+^. Chemical Formula: C_24_H_23_N_5_O_5_; Calcd. For: C, 62.47; H, 5.02; N, 15.18; Found: C, 62.49; H, 5.06; N, 15.22.

*(E)-3-(3,4-Dimethoxyphenyl)-N-(4-(1,3,7-trimethyl-2,6-dioxo-2,3,6,7-tetrahydro-1H-purin-8-yl)phenyl)-acrylamide* (**PX-E-M10**). Dark brown solid, Yield: 46.3%; mp > 300 °C; IR (cm^−1^): ν_N-H_: 3525.19, 3283.08; ν_C=O_: 1681.79, 1645.73, 1625.04. ^1^H-NMR (DMSO-d_6_): 3.26 (s, 3H, N-CH_3_), 3.38 (s, 3H, N-CH_3_), 3.48 (s, 3H, N-CH_3_), 3.82 (s, 3H, -OCH_3_), 4.02 (s, 3H, -OCH_3_), 6.72 (d, 1H, *J* = 15.6 Hz, 6′-PhH), 7.03 (d, 1H, *J* = 7.4 Hz, CONH-PhH), 7.19 (d, 1H, *J* = 7.4 Hz, CONH-PhH), 7.22 (d, 1H, *J* = 8.4 Hz, COCH=CH), 7.51 (t, 1H, *J* = 7.4 Hz, CONH-PhH), 7.53 (d, 1H, *J* = 8.4 Hz, COCH), 7.55 (d, 1H, *J* = 15.6 Hz, 5′-PhH), 7.87 (s, 1H, CONH-PhH), 8.17 (s, 1H, 2′-PhH), 10.38 (s, 1H, CONH). EI-MS: 476.1 [M + H]^+^. Chemical Formula: C_25_H_25_N_5_O_5_; Calcd. For: C, 63.15; H, 5.30; N, 14.73; Found: C, 63.26; H, 5.34; N, 14.69.

### 3.3. Affinity Toward A2AR

The binding affinity of test compounds was determined using radioligand binding assays. Human A2AR cDNA was stably transfected into HEK-293 cells. Membranes from the transfected cell were used in the interactive capacity of each compound toward the A2AR. Competition binding assay experiments by incubating membranes of 10 μg protein with 2 nM [3H]ZM241385 were carried out, with interval concentrations ranging from 0.1 nM to 100 μM of test compounds in 96-well filter plates at 4 °C for 90 min. After incubation experiments, free and bound radioligands were split by filtering and washing. Last, filter-bound radioactivity was counted after addition of 30 μL/well of scintillation solution. Nonspecific binding was measured in the presence of 100 μM ZM241385. The experiments were carried in duplicate. IC_50_ values were determined using a nonlinear regression curve fitting by the program GraphPad Prism. The affinities of compounds were calculated by Ki value which was from the IC_50_ values according as the Cheng−Prusoff equation [19].

### 3.4. MAO-B Inhibition Studies

Inhibitory potency on MAO-B enzyme from recombinant human MAO-B were carried out by a fluorometric method with a commercial kit (Promega, MAO-Glo Assay) [18]. The MAO enzyme incubation experiments were carried with a MAO-B substrate, methyl (*S*)-2-(6-(2-aminoethoxy) benzo[d]thiazol-2-yl)-4,5-dihydrothiazole-4-carboxylate, which was converted to luciferin methyl ester. Then the “Luciferin Detection Reagent” was added to change the methyl ester derivative to luciferin, stop the MAO-B reaction, and simultaneously produce light. The intensity of light is directly in proportion to MAO-B activity. The assay was carried in a 96-well black opaque microplate in duplicate. The reaction mixture was composed of 12.5 μL of MAO-B substrate (a final concentration of 4 μM), 25 μL of human MAO-B (1 μg of protein/well), and 12.5 μL of test compounds for each well. After 1 h of incubation at 37 °C, each well added 50 μL of “Luciferin Detection Reagent”, and incubated an additional 20 min. the luminescent signal of microplate was measured by a luminometer. The signal from reaction of MAO-B enzyme and substrate without inhibitors accounted as the positive control, whereas the signal from reaction of MAO-B enzyme and inhibitors in the absence of substrate accounted as the negative control. Compound concentrations of 1 μM were tested at first. IC_50_ values (the half maximal inhibitory concentration) were performed from concentration–response curves, plotted by the GraphPad Prism software. All experiments were performed in duplicate, and data are expressed as mean ± SD.

### 3.5. MTT Cytotoxicity Assay

The MTT assay method is a colorimetric assay which is based on cell metabolic activity, whereby active cells can reduce MTT [3,[3-(4,5-dimethylthiazol-2-yl)-2,5-diphenyltetrazolium bromide] to its purple formazan which can be measured by spectrophotometry. The antiproliferative activity of cytotoxicity was determined using an MTT assay. The cytotoxic activity of test compounds was assessed in human neuroblastoma SH-SY5Y cell Line. In brief, 1 × 10^5^ cells per well were plated in 96-well culture plates in quadruplicate and incubated for 24 h in a CO_2_ incubator. Test compounds were added to the wells with 10μM final concentration. After 24 h of cells incubation with test compounds, 10 μL of MTT labeling reagent was added in each well and then incubated for 4 h at 37 °C. Following incubation, supernatant was taken away from each well and 100 μL of DMSO reagent was added to each well. The absorbance of each well was measured by at 570 nm after 15min delay. The percentage antiproliferative activities were calculated by using the formula: Viability (%) = {[A570 (sample absorbance) − (blank absorbance)]/[A570 (control absorbance) − blank absorbance]} × 100. Cells treated with solvent DMSO were used as the control. The media free of cells was used as the blank sample. 

### 3.6. Pharmacokinetic Experiments 

Sprague-Dawley rats (200 ± 20 g) were obtained from the Laboratory Animal Center of Wenzhou Medical University (Wenzhou, China) to study the pharmacokinetics and tissue distribution studies (TDS) of **PX-D-P6** and **PX-E-P8** following intraperitoneal injection (i.p.) administration. The experiment procedures and protocols were approved the Animal Care and Use Committee of Wenzhou Medical College and complied with the Guide for the Care and Use of Laboratory Animals. The corresponding ethical approval code was WMU20161025. 

The chosen compounds dissolved in suspension formulation (2 % Tween 80–saline) were administered to each rat at a dose of 50 mg/kg. Blood samples (0.3 mL) were collected from the caudal vein into 1.5 mL tapered plastic centrifuge tubes (pretreated with K_2_EDTA as an anticoagulant) at pre-dose, 0.08, 0.16, 0.25, 0.5, 1, 2, 4, 8, 12, 24 h after i.p. administration. Separately, blood and brain samples were collected at pre-dose and 1 h for TDS. Blood samples were immediately centrifuged at 3000 g for 10 min for isolation of the plasma. Brain samples were homogenized in phosphate buffer saline. 100 μL plasma or 200 μL brain sample was precipitated with addition of 400 μL acetonitrile and vortex mixed for 1 min and centrifuged at 10,000 g for 10 min. Finally, 2μL supernatant was injected into UPLC/MS system. Calibration standards samples were prepared with blank rat plasma or brain homogenates. Plasma pharmacokinetic parameters were analyzed by DAS (Drug and Statistics) software (Version 2, Wenzhou Medical University, Wenzhou, China).

### 3.7. Pharmacological Potential: Catalepsy

Catalepsy was measured using a thin metal bar test at 9.0 cm height [23]. In this test, each rat was injected intraperitoneally by haloperidol (1.5 mg/kg). Before i.p. administration of vehicle (2% Tween 80–saline), **PX-D-P6** and **PX-E-P8** (5, 15, 50 mg/kg), at time 0 min, we tested all rats for successful induction of catalepsy. The length of time (descent latency) it persisted in hanging onto the metal bar was measured for a period of up to 300 s (maximum score). After this period, the rat was gently removed from the bar. The high bar test was performed 20 min after the injection of compound. Descent latencies have been presented as median and interquartile ranges. Differences between medians were analyzed by Kruskal-Wallis test, followed by Bonferroni's test. 

## 4. Conclusions

In this work, a series of PX derivatives were synthesized and evaluated for their antiparkinsonian properties. On the whole, PX-D analogues showed slightly better antiparkinsonian properties than PX-E analogues. Of all the compounds, **PX-D-P6** and **PX-E-P8** were verified as potent A2AR antagonists and MAO-B inhibitors with desirable drug-like properties, including good pharmacokinetic parameters, brain penetration and anti-catalepsy properties. We thus found that PX-D and PX-E analogues are promising candidate dual-acting drugs with antiparkinsonian properties.

## Figures and Tables

**Table 1:** Biological data and structure−activity relationship (SAR) of A2AR affinities (Ki) and MAO-B inhibitory potencies (Ki) for test compounds.

Compound	-HNCO	R_1_	R_2_	R_3_	Ki hA2AR (μM) ^1^	Ki hMAO-B (μM) ^1^
Istradefylline					0.05 ± 0.01	>10
**PX-D-P1**	*para-*	H	H	H	2.31 ± 0.31	1.32 ± 0.11
**PX-D-P2**	*para-*	H	H	CH_3_	2.87 ± 0.21	2.06 ± 0.25
**PX-D-P3**	*para-*	H	H	OCH_3_	2.67 ± 0.14	2.94 ± 0.31
**PX-D-P4**	*para-*	H	Cl	H	1.52 ± 0.16	0.25 ± 0.05
**PX-D-P5**	*para-*	H	OCH_3_	OCH_3_	4.27 ± 0.51	1.92 ± 0.25
**PX-D-P6**	*para-*	CH_3_	H	H	0.33 ± 0.09	0.29 ± 0.03
**PX-D-P7**	*para-*	CH_3_	H	CH_3_	0.39 ± 0.06	1.21 ± 0.15
**PX-D-P8**	*para-*	CH_3_	H	OCH_3_	1.19 ± 0.12	0.47 ± 0.12
**PX-D-P9**	*para-*	CH_3_	Cl	H	0.27 ± 0.08	1.51 ± 0.19
**PX-D-P10**	*para-*	CH_3_	OCH_3_	OCH_3_	1.34 ± 0.16	2.97 ± 0.37
**PX-D-M1**	*meta-*	H	H	H	2.91 ± 0.21	3.11 ± 0.42
**PX-D-M2**	*meta-*	H	H	CH_3_	4.51 ± 0.57	2.17 ± 0.29
**PX-D-M 3**	*meta-*	H	H	OCH_3_	3.77 ± 0.55	2.57 ± 0.37
**PX-D-M 4**	*meta-*	H	Cl	H	3.61 ± 0.41	1.27 ± 0.18
**PX-D-M5**	*meta-*	H	OCH_3_	OCH_3_	4.99 ± 0.75	2.36 ± 0.31
**PX-D-M6**	*meta-*	CH_3_	H	H	2.03 ± 0.25	2.26 ± 0.29
**PX-D-M7**	*meta-*	CH_3_	H	CH_3_	2.78 ± 0.33	2.57 ± 0.31
**PX-D-M8**	*meta-*	CH_3_	H	OCH_3_	3.05 ± 0.36	2.86 ± 0.21
**PX-D-M9**	*meta-*	CH_3_	Cl	H	2.44 ± 0.21	0.76 ± 0.11
**PX-D-M10**	*meta-*	CH_3_	OCH_3_	OCH_3_	3.59 ± 0.36	2.54 ± 0.31
**PX-E-P1**	*para-*	H	H	H	4.97 ± 0.59	8.11 ± 1.03
**PX-E-P2**	*para-*	H	H	F	3.41 ± 0.47	9.36 ± 1.21
**PX-E-P3**	*para-*	H	CF_3_	H	5.18 ± 0.67	4.22 ± 0.55
**PX-E-P4**	*para-*	H	Cl	H	6.43 ± 0.98	1.75 ± 0.15
**PX-E-P5**	*para-*	CH_3_	H	H	1.46 ± 0.17	>10
**PX-E-P6**	*para-*	CH_3_	H	F	0.79 ± 0.11	5.04 ± 0.53
**PX-E-P7**	*para-*	CH_3_	CF_3_	H	1.98 ± 0.15	>10
**PX-E-P8**	*para-*	CH_3_	Cl	H	0.85 ± 0.11	0.63 ± 0.11
**PX-E-M1**	*meta-*	H	H	H	9.65 ± 0.98	3.42 ± 0.32
**PX-E-M2**	*meta-*	H	H	F	7.23 ± 0.86	2.48 ± 0.23
**PX-E-M3**	*meta-*	H	CF_3_	H	7.44 ± 0.91	2.63 ± 0.31
**PX-E-M4**	*meta-*	H	Cl	H	9.58 ± 1.13	>10
**PX-E-M5**	*meta-*	H	OCH_3_	OCH_3_	>10	2.11 ± 0.19
**PX-E-M6**	*meta-*	CH_3_	H	H	5.25 ± 0.65	3.72 ± 0.52
**PX-E-M7**	*meta-*	CH_3_	H	F	7.74 ± 0.92	2.58 ± 0.37
**PX-E-M8**	*meta-*	CH_3_	CF_3_	H	7.1 ± 0.84	4.76 ± 0.68
**PX-E-M9**	*meta-*	CH_3_	Cl	H	9.18 ± 1.23	2.93 ± 0.41
**PX-E-M10**	*meta-*	CH_3_	OCH_3_	OCH_3_	6.24 ± 0.77	2.55 ± 0.24

^1^ Ki values were converted from IC_50_ values, by means of the method of Cheng and Prusoff. The determinations were performed in duplicate and the values are expressed as mean ± SD.

**Table 2:** The main pharmacokinetic parameters after i.p. administration of 50 mg/kg dose in rats (*n* = 6).

Parameters	PX-D-P6	PX-E-P8
T_max_ (h)	0.51 ± 0.15	0.45 ± 0.21
C_max_ (ng/mL)	2112.6 ± 508.4	1642.1 ± 435.9
t_1/2_ (h)	4.51 ± 0.61	3.27 ± 0.39
AUC0→t (ng/mL·h)	30526.2 ± 1206.7	15564.2 ± 1537.4
CL (L/h/kg)	5.47± 1.23	10.28 ± 2.19

**Table 3:** Plasma and brain assays for **PX-D-P6** and **PX-E-P8** at 1 h after i.p. 50 mg/kg dose in rats (*n* = 6).

Compound	Plasma (ng/mL)	Brain (ng/g)	B/P Ratio
**PX-D-P6**	1126 ± 207	1289 ± 132	1.14
**PX-E-P8**	915 ± 231	887 ± 113	0.97
